# Supplementary material for: The discovery of novel noncoding RNAs in 50 bacterial genomes
Source: Nucleic Acids Res. 2024 Apr 22;52(9):5152–65. doi: 10.1093/nar/gkae248 (PMC11109978; doi:10.1093/nar/gkae248)
Supplement: gkae248_Supplemental_Files [file gkae248_supplemental_files.zip › Narunsky 50 Genomes RNA Motifs Files.zip!/Publication/46-B_bacilliformis/Figures/LRC-46-7-NC_008783_1_IGR_34_229189_229423.sto.pdf]

LRC-46-7-NC_008783_1_IGR_34_229189_229423.sto.r2r

5´-GARAUGRU●CYGGGUGCUAUGCAARU○CCAUAURAGUACRRGCCAAGAG●GRGGCR○○○○○○G●ARGAGGUAUGGGUYAAGAGCCRUGAYGCC○○○○○○●R●GGGGUYA●GGG●C●A●G●AGARGCY○
